# *Allium hookeri* root protects oxidative stress-induced inflammatory responses and β-cell damage in pancreas of streptozotocin-induced diabetic rats

**DOI:** 10.1186/s12906-016-1032-1

**Published:** 2016-02-17

**Authors:** Seong-Soo Roh, O Jun Kwon, Jae Heon Yang, You Suk Kim, Sung Hyun Lee, Jong-Sik Jin, Yong-Deok Jeon, Takako Yokozawa, Hyun Ju Kim

**Affiliations:** College of Korean Medicine, Daegu Haany University, Daegu, 706-060 Republic of Korea; Gyeongbuk Regional industry Evalution, Daegyeong Institute for Regional Program Evalution, Gyeongsan-si, 712-210 Republic of Korea; Center for Healthcare Technology Development, Chonbuk National University, Jeonju-si, 595-831 Republic of Korea; Sunchang County Agricultural Research Center, Sunchang-gun, 595-831 Republic of Korea; Department of Agro-food Resource, Rural Development Administration, Jeonju-si, 565-851 Republic of Korea; Department of Oriental Medicine Resources, Chonbuk National University, Iksan-si, 570-752 Republic of Korea; Graduate School of Science and Engineering for Research, University of Toyama, Toyama, Japan; Microbiology and Functionality Research Group, World Institute of Kimchi, Gwangju, 61755 Republic of Korea

**Keywords:** *Allium hookeri*, Oxidative stress, Inflammation, Pancreas, Streptozotocin-induced diabetes

## Abstract

**Background:**

Water extract from the root of *Allium hookeri* (AH) shows anti-inflammatory, antioxidant, and free radical scavenging effects. In this study, the ameliorating effects of AH on oxidative stress-induced inflammatory response and β-cell damage in the pancreas of streptozotocin (STZ)-induced type 1 diabetic rats were investigated.

**Methods:**

AH (100 mg/kg body weight/day) was orally administered every day for 2 weeks to STZ-induced diabetic rats. After the final administration of AH, biochemical parameters including glucose, insulin, reactive oxygen species levels, and protein expressions related to antioxidant defense system in the pancreas of STZ-induced diabetic rats.

**Results:**

The diabetic rats showed loss of body weight and increased pancreatic weight, while the oral administration of AH attenuated body and pancreatic weight changes. Moreover, the administration of AH caused a slightly decrease in the serum glucose level and a significant increase in the serum and pancreatic insulin levels in the diabetic rats. AH also significantly reduced the enhanced levels of reactive oxygen species, oxidative stress biomarker, in the serum and pancreas. The diabetic rats exhibited a down-regulation of the protein expression related to antioxidant defense system in the pancreas, but AH administration significantly up-regulated the expression of the heme oxygenase-1 (HO-1). Furthermore, AH treatment was reduced the overexpression of nuclear factor-kappa B (NF-кB)p65 and NF-кBp65-induced inflammatory cytokines such as tumor necrosis factor-α and interleukin-6. In addition, AH treatment was less pancreatic β-cell damaged compared with those of the diabetic rats.

**Conclusion:**

These results provide important evidence that AH has a HO-1 activity on the oxidative stress conditions showing pancreato-protective effects against the development of inflammation in the diabetic rats. This study provides scientific evidence that AH protects the inflammatory responses by modulated NF-кBp65 signaling pathway through activation of HO-1 in the pancreas of STZ-induced diabetic rats.

## Background

Type 1 diabetes, which is the chronic autoimmune disease, results from the destruction of the insulin-producing β-cell of the pancreas, leading to a gradual decrease in β-cell mass [1]. Accordingly, type 1 diabetes shows absolute insulin deficiency and excessive glucose production [2, 3]. Hyperglycemia is the main clinical symptom of type 1 diabetes, which causes glycation of body proteins that in turn leads to secondary complications affecting the eyes, kidneys, nerves and blood vessels. Standard diabetes management for type 1 diabetes is based on exogenous insulin replacements. However, this treatment leads to frequently the severe hypoglycemic state [4]. Also, the severity of type 1 diabetes is progressively increasing associated with various complications either at the macrovascular level causing coronary artery and cerebrovascular diseases or at the microvascular level causing renal failure, blindness, limb amputation, neurological complications, and premature death [5]. Thus, new type of therapies needs to decrease blood glucose levels, affects pancreatic β-cell and blood glucose. Recently, diverse studies are reported that increased oxidative stress induced by hyperglycemia is associated with type 1 diabetes [6–8].

Oxidative stress can be defined as an imbalance between the production of some highly reactive molecule species and the antioxidant defense system [9]. In the process of oxidative stress, excessive reactive oxygen species (ROS) were produced, mainly by mitochondria. ROS or free oxygen radicals are products of normal cellular metabolism; however, their unbalanced increased levels disrupt normal cellular function. The most common free oxygen radicals are hydroxyl radical (∙OH), nitric oxide (NO), superoxide (O_2_^−^), hydrogen peroxide (H_2_O_2_), and peroxynitrite (ONOO^−^) [10, 11]. In fact, the overproduction of free radicals have been related to cellular membrane, protein, RNA and DNA damage, and indirectly with aging and oxidative stress-related diseases like cancer, cardiovascular, inflammatory, and neurodegenerative pathologies [12]. Eventually, they could cause damage and apoptosis of pancreatic islet β-cells and reduction of insulin secretion. Thus, the antioxidant therapy has gained an utmost importance in the treatment of such diseases linked to free radicals [13]. Recently, research on herbal medicine without limited or side effects has been studied as an alternative medicine for treating diabetes by antioxidant effect [14, 15].

*Allium hookeri* Thwaites (Liliaceae family, AH) is widely distributed in India, Sri Lanka, Myanmar, Bhutan, and southwestern China [16]. Recently, this plant was introduced to South Korea and has been cultivated in the southern region [17]. Genus *Alliums* produces chemical compounds known cystein sulfoxide and these sulfur containing compounds give them a specific feature onion (*Allium cepa*) or garlic (*Allium sativum* L.) taste and smell [18]. Sulfur is an important component as parts of the essential amino acids cysteine and methionine, which forms a protein block in connective tissues and muscle. Sulfur protects cells from damage due to radical activity such as oxidation, because thiols formed in the body are related to reduce oxidation [19]. However, sulfur should not be eaten directly due to side effects; therefore, sulfur should be taken indirectly in food with a high sulfur concentration. Thus, AH has been used as food and the recipes generally are prepared by frying, steaming, baking and boiling. In southeast Asia, the root of AH has been added in Kimchi, which is a traditional fermented Korean foodmade of vegetables with a variety of seasonings [20]. The root of AH also has some medicinal values. They are used for treating cold and cough, for healing burn injuries and wounds. Recently, some reports showed anti-inflammatory, antioxidant, and free radical scavenging effects [21–23]. Additionally, previous study demonstrated that metanolic extract of AH leaf exhibited anti-diabetic activity such as reductions of blood glucose and lipid parameters in streptozotocin (STZ)-inuced type 1 diabetic rats [24]. In our previous study, diet of AH leaf or root showed lower blood glucose and HbA1c levels, and increased density of immunoreactives cells in the type 2 diabetic *db/db* mice [25]. Therefore, this study decided to clarify the detailed mechanisms involved in anti-diabetic action of AH through its effect on oxidative stress-induced inflammatory response in the pancreas of the STZ-induced diabetic rats.

## Methods

### Materials

**Fig. 1:**
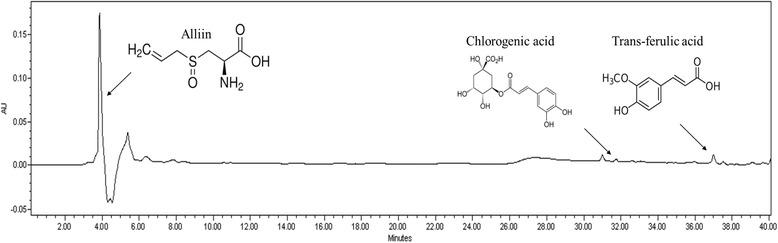
Identification of chemical ingredients of root extract of *Allium hookeri*

(±)-L-Alliin, trans-ferulic acid and chlorogenic acid (Fig. 1) were purchased from Sigma-Aldrich (St. Louis, USA). Diethylenetriaminepentaacetic acid (DTPA), dihydrorhodamine 123 (DHR 123), and phenylmethylsulfonyl fluoride (PMSF) were obtained from Sigma-Chemical Co. (St. Louis, MO, USA). 2’,7’-Dichlorofluorescein diacetate (DCFH-DA) was obtained from Molecular Probes (Eugene, OR, USA). The protease inhibitor mixture and ethylenediaminetetraacetic acid (EDTA) were purchased from Wako Pure Chemical Industries, Ltd. (Osaka, Japan). BCA protein assay kit (bicinchoninic acid) was obtained from Thermo Scientific (Rockford, IL, USA). Rabbit polyclonal antibodies against superoxide dismutase (SOD), catalase, heme oxygenase-1 (HO-1) and NF-кBp65, goat polyclonal antibodies against tumor necrosis factor-α (TNF-α) and interleukin-6 (IL-6), and mouse monoclonal antibodies against β-actin and histone were purchased from Santa Cruz Biotechnology, Inc. (Santa Cruz, CA, USA). Goat anti-rabbit and goat anti-mouse immunoglobulin G (IgG) horseradish peroxidase (HRP)-conjugated secondary antibodies were acquired from Santa Cruz Biotechnology, Inc. ECL Western Blotting Detection Reagents were supplied by GE Healthcare (Piscataway, NJ, USA).

### Plant material

*Allium hookeri* was cultivated and harvested in Sunchang (Korea). *Allium hookeri* Thwaites was listed on The Plant List based on database of World Checklist of Selected Plant Families in 2012 (http://www.theplantlist.org). Dried *Allium hookeri* root was suspended with ten volumes of distilled water and extracted for 12 h in a water bath at 95 °C. The residues were reextracted three times under the same conditions. The hot-water extracts were filtered through Whatman filter paper (grade No. 2; Whatman International, Kent, UK), freeze-dried, pulverized, and stored at −40 °C until further analysis. A voucher specimen was deposited in the herbarium of the World Institute of Kimchi.

### Identification of chemical ingredients of root extracts of *Allium hookeri* with HPLC

Root extract of *Allium hookeri* was dissolved in MeOH into 50 mg/ml amd 20 ul was injected. Alliin, trans-ferulic acid, and chlorogenic acid were dissolved in MeOH into 1 mg/ml for HPLC analysis. HPLC was performed on a Waters 2695 separation module (Waters, Milford, USA) with Waters 2487 dual λ absorbance detector (Waters, Milford, USA) under following condition: column, TSK-gel ODS-80Ts (Tosoh Co., Tokyo, Japan 4.6 mmx150 mm); mobile phase, 0.1 % formic acid (solvent system A) and CH_3_CN (solvent system B) in a gradient mode (B 5 % from 0 to 20 min, B from 5 to 50 % from 20 to 40 min); flow rate, 0.5 ml/min; temperature, 30 °C; UV wavelength, 254 nm.

### Experimental animals and treatment

Animal experiments were performed according to the laboratory conditions [26] of the Daegu Haany University (Daegu Haany University 2013–036). Six-week-old male Sprague–Dawley rats were purchased from Daehan-Bio (Chungcheong, Korea). The rats were maintained under a 12-h light/dark cycle, and housed in a controlled temperature (24 ± 2 °C) and humidity (50 ± 5 %) environment. After several days of adaptation, the rats were divided into normal and diabetic groups. The diabetic group was injected intraperitoneally with STZ (Sigma-Aldrich Co.) (50 mg/kg body weight) in a 10 mM citrate buffer (pH 4.5) [27]. The blood glucose level determined and the body weight was measured 10 days after the injection, and to avoid any intergroup differences in these indices, the rats were divided into two experimental groups: group 1, diabetic rats received water (DC, *n* = 5); group 2, diabetic rats received 100 mg/kg body weight/day of AH (*n* = 5) orally through gavage once a day, respectively. The administration dose and duration were determined based on previous study [25] and preliminary data. Data showed that an oral glucose tolerance at 30 to 120 min was significantly improved in the AH 100 mg/kg B.W. treated groups, compared to the normal group (p < 0.05, data not shown). The rats that underwent a sham injection of citrate buffer without STZ were also used as a nondiabetic group (NC, *n* = 5). After 2 weeks of treatment, blood samples were obtained from the abdominal aorta under pentobarbital anesthesia (50 mg/kg body weight, intraperitoneally), and serum was separated immediately by centrifugation. Subsequently, each rat was perfused with ice-cold physiological saline, and then the pancreas was removed, snap-frozen in liquid nitrogen, and stored at −80 °C until analyses.

### Measurement of glucose and insulin in the serum

The serum glucose level was examined using a commercial kit (Asan Pharm. Co., Ltd., Hwaseong-si, Korea), and serum insulin level was measured by ELISA kit (EMD Millipore, Billerica, MA, USA).

### Measurement of ROS in the serum and pancreas

ROS level was measured employing the method of Ali et al. [28]. Pancreatic tissue was homogenized on ice with 1 mM EDTA-50 mM sodium phosphate buffer (pH 7.4), and then 25 mM DCFH-DA was added to homogenates or serum. After incubation for 30 min, the changes in fluorescence values were determined at an excitation wavelength of 486 nm and emission wavelength of 530 nm.

### Preparation of nuclear and postnuclear fractions

Nuclear protein extraction was performed according to the method of Komatsu [29]. In brief, pancreatic tissue was homogenized with ice-cold lysis buffer containing 5 mM Tris–HCl (pH 7.5), 2 mM MgCl_2_, 15 mM CaCl_2_, and 1.5 M sucrose, and then 0.1 M dithiothreitol (DTT) and protease inhibitor mixture were added. After centrifugation (10,500 × g for 20 min at 4 °C), the pellet was suspended with extraction buffer containing 20 mM 2-[4-(2-hydroxyethyl)-1-piperazyl] ethanesulfonic acid (pH 7.9), 1.5 mM MgCl_2_, 0.42 M NaCl, 0.2 mM EDTA, and 25 % (v/v) glycerol, and then 0.1 M DTT and protease inhibitor mixture were added. The mixture was placed on ice for 30 min. The nuclear fraction was prepared by centrifugation at 20,500 × g for 5 min at 4 °C. The post-nuclear fraction was extracted from the pancreas of each rat, as described below. In brief, pancreatic tissue was homogenized with ice-cold lysis buffer (pH 7.4) containing 137 mM NaCl, 20 mM Tris–HCl, 1 % Tween 20, 10 % glycerol, 1 mM PMSF, and protease inhibitor mixture. The homogenate was then centrifuged at 2,000 × g for 10 min at 4 °C. The protein concentration in each fraction was determined using a pierce bicinchoninic acid (BCA) protein assay kit (Thermo Scientific, Rockford, IL, USA).

### Immunoblotting analyses

For the determination of NF-кBp65 and histone, 10 μg of protein from each nuclear fraction was electrophoresed through a 10 % sodium dodecylsulfate polyacrylamide gel (SDS-PAGE). Separated proteins were transferred to a nitrocellulose membrane, blocked with 5 % (w/v) skim milk solution for 1 h, and then incubated with primary antibodies to NF-кBp65 and histone overnight at 4 °C. After the blots were washed, they were incubated with anti-rabbit or anti-mouse IgG HRP-conjugated secondary antibody for 1 h at room temperature. Also, 10–15 μg of protein of each postnuclear fraction of SOD, catalase, HO-1, TNF-α, IL-6, and β-actin was electrophoresed through 12 % SDS-PAGE. Each antigen-antibody complex was visualized using ECL Western Blotting Detection Reagents and detected by chemiluminescence with Sensi-Q 2000 (Lugen Sci., Gyeonggi-do, Korea). Band densities were determined using ATTO Densitograph Software (ATTO Corporation, Tokyo, Japan) and quantified as the ratio to histone or β-actin. The protein levels of groups are expressed relative to those of normal rats (represented as 1).

### Histological examination of pancreatic tissue

For microscopic evaluation, the pancreas was cut to isolate the middle segment. This segment was fixed in 10 % neutral-buffered formalin and after embedding in paraffin, cut into 2 μm sections and stained using hematoxylin and eosin (H/E) for microscopic evaluation. The stained slices were subsequently observed under an optical microscope and analyzed using the i-Solution Lite software program (Innerview Co., Ltd., Seongnam-si, Korea).

### Statistical analysis

Data are expressed as means ± SEM. Significance was assessed by one-way analysis of variance (ANOVA) followed by Dunnett's multiple comparison test (SPSS 11.5.1 for Windows, 2002, SPSS Inc., USA). Values of *p* < 0.05 were considered significant.

## Results

### Identification of chemical ingredients of root extract of *Allium hookeri*

When alliin, chlorogenic acid, and tran-ferulic acid were analyzed with HPLC, the retention times were 3.8, 31.9, and 37.0 min. As shown in Fig. 1, the compounds were detected in water extract of *Allium hookeri* root.

### Body weight, food intake, water intake, and pancreatic weight

Table 1 shows the changes in the body weight, food intake, water intake, and pancreatic weight during the experimental period. The diabetic control rats displayed a marked decrease in body weight, and the decreased body weight was significantly increased by AH-treated diabetic rats. The food and water intakes were not changed by AH treatment. The pancreatic weight in diabetic control rats was 2.1 times greater than that in non-diabetic rats, but was significantly decreased by AH administration.

**Table 1:** Body weight, food intake, water intake, and pancreatic weight

Groups	Body weight			Food intake	Water intake	Pancreas weight
	Initial (g)	Final (g)	Change (g/15 days)	(g/day)	(ml/day)	(mg/100 g body weight)
NC	367 ± 5^***^	411 ± 6^***^	44 ± 5^***^	23 ± 1^***^	28 ± 1^***^	4.6 ± 0.2^***^
DC	260 ± 8	225 ± 9	−35 ± 3	38 ± 1	212 ± 6	9.7 ± 0.3
AH	275 ± 9	256 ± 10^*^	−19 ± 3^**^	38 ± 2	211 ± 11	6.7 ± 0.4^***^

NC, nondiabetic control rats; DC, diabetic control rats; AH, diabetic AH-treated rats. Data are the mean ± SEM. *P < 0.05, **P < 0.01, ***P < 0.001 versus DC

### Serum glucose, insulin, and ROS levels

As shown in Table 2, the serum glucose level in diabetic control rats was markedly higher than that in non-diabetic rats, but it was slightly decreased in AH-administered group. The insulin level was significantly lower in the diabetic than that in non-diabetic rats. Treatment with AH increased significantly this level. The level of ROS, oxidative stress biomarker, in the serum of diabetic control rats was significantly elevated compared with non-diabetic rats; however, this level on receiving AH was significantly decreased.

**Table 2:** Serum glucose, insulin and ROS levels

Group	Glucose	Insulin	ROS
	(mg/dl)	(ng/ml)	(fluorescence/min/ml)
NC	130 ± 1^**^	1.00 ± 0.01^**^	45 ± 7^**^
DC	412 ± 2	0.40 ± 0.07	310 ± 29
AH	386 ± 26	0.67 ± 0.01^**^	201 ± 26^*^

*NC*, nondiabetic control rats; *DC*, diabetic control rats; *AH*, diabetic AH-treated rats

Data are the mean ± SEM. *P < 0.05, **P < 0.001 versus DC

### Pancreatic insulin content

**Fig. 2:**
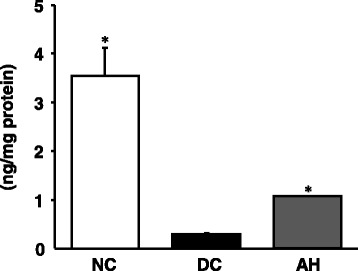
Insulin expression in the pancreas. NC, non-diabetic control rats; DC, diabetic control rats; AH, *Allium hookeri*-treated diabetic rats. The results are presented as the mean ± SEM for five rats in each group. ^*^ *P* < 0.001 versus DC

Concerning the insulin content, the diabetic control rats showed a marked decrease compared with non-diabetic control rats. This content was significantly increased by AH-administered rats (*p* < 0.001), as shown in Fig. 2.

### Pancreatic ROS content

**Fig 3.**
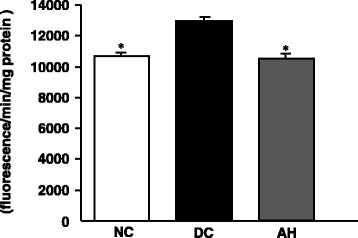
ROS content in the pancreas. NC, non-diabetic control rats; DC, diabetic control rats; AH, *Allium hookeri*-treated diabetic rats. The results are presented as the mean ± SEM for five rats in each group. ^*^ *P* < 0.001 versus DC

As shown in Fig. 3, the content of ROS in the pancreas of diabetic control rats was significantly increased compared to that of non-diabetic rats, whereas this elevated content was significantly decreased nearly to the content of non-diabetic rats in AH-administered diabetic rats.

### Pancreatic antioxidant enzyme-related protein expressions

**Fig. 4:**
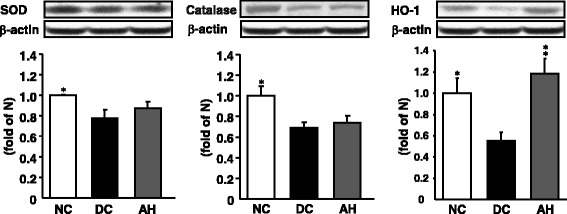
Representative SOD, catalase, and HO-1 protein expressions in the pancreas. NC, non-diabetic control rats; DC, diabetic control rats; AH, *Allium hookeri*-treated diabetic rats. The results are presented as the mean ± SEM for five rats in each group. ^*^ *P* < 0.05, ^**^ *P* < 0.01 versus DC

The expression levels of antioxidant enzyme-related proteins, such as SOD, catalase and HO-1, in diabetic control rats were significantly lower than those of non-diabetic rats, as shown in Fig. 4. The decreased protein expressions of SOD and catalase were slightly increased, not significantly, by the AH administration. On the contrary, the decreased protein expression of HO-1 was increased to nearly non-diabetic level in the AH-administered diabetic rats.

### Pancreatic inflammation-related protein expressions

**Fig. 5:**
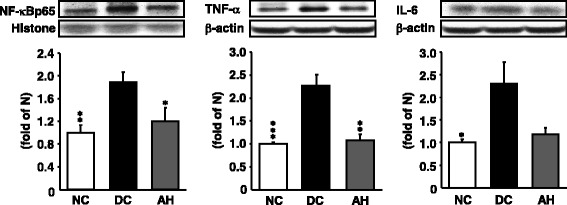
Representative NF-кBp65, TNF-α, and IL-6 protein expressions in the pancreas. NC, non-diabetic control rats; DC, diabetic control rats; AH, *Allium hookeri*-treated diabetic rats. The results are presented as the mean ± SEM for five rats in each group. ^*^ *P* < 0.05, ^**^ *P* < 0.01, ^***^ *P* < 0.001 versus DC

The pancreas of diabetic rats showed the up-regulation of NF-кBp65 protein and NF-кB-induced pro-inflammatory protein (TNF-α and IL-6) expression levels compared with non-diabetic rats; however, the administration of AH to diabetic rats decreased the expressions of these inflammation-related proteins nearly to the level of non-diabetic rats (Fig. 5).

### Pancreatic histological examination

**Fig. 6:**
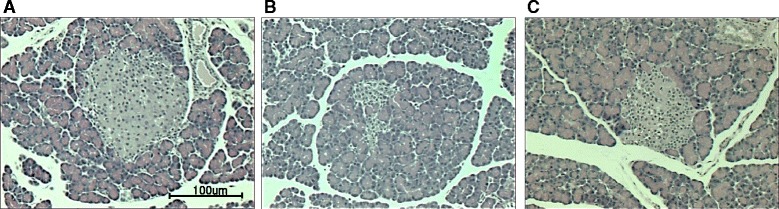
H/E staining of pancreatic tissue. **a** non-diabetic control rats, **b** diabetic control rats, **c** *Allium hookeri*-treated diabetic rats. Images are at × 400 magnification (n = 5)

In the histological examinations using HE staining, a decrease of pancreatic islet numbers and size was exhibited in the pancreatic tissue of diabetic control rats compared with non-diabetic rats, as shown in Fig. 5(a) and (b). AH administration group distinctly improved these abnormal histological phenomena (Fig. 6(c)).

**Fig. 7:**
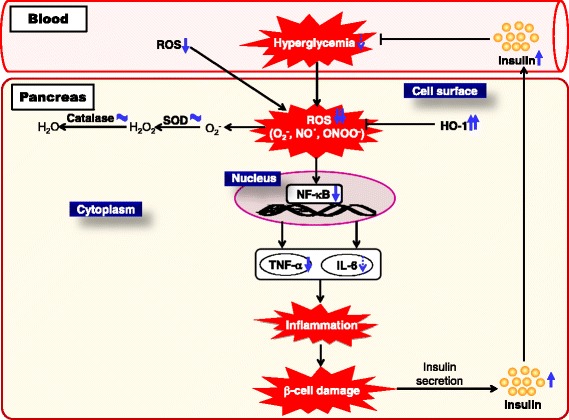
Predicted mechanism in pancreatic tissue on administering AH. Our study revealed that AHR suppresses STZ-induced type 1 diabetes. An important mechanism of AHR’s anti-diabetic effect is its capacity to reduce the oxidative stress state by diminishing ROS generation and lipid peroxidation in the pancreas. Our data further suggest that another critical mechanism of AHR’s anti-diabetic property is its ability to ameliorate inflammation through modulation of the serum TNF-α and IL-6 levels and the pancreatic protein expressions of NF-кB

## Discussion

AH, a wild herb growing in a wide range soils, distributed in India and Myanmar. Recently, AH which is cultivated in South Korea has been used as food and medicine. Unlike onion or any other *Allium* species, AH has hardly any bulb and produces fibrous roots, instead. AH contains protein, sugar, ascorbic acid, phytosterols, and total phenols. Phenolic component inhibits the radicals and decreases the side effects on normal physiological functions [30]. Especially, it has been reported that AH contained allicin and ten alkyl thiosulfinates by HPLC-electrospray ionization-mass spectrometry (HPLC-ESI-MS) [17]. In addition, AH of South Korea contained twice the amount of sulfur and crude saponin, relative to AH of Myanmar in comparison with the study of nutrient composition and quality of AH between South Korea and Myanmar [31]. Also, the main medical activities of AH are anti-inflammatory, antioxidant, and free radical scavenging effects. However, there has been no study whether AH has an ameliorative effect on oxidative stress-induced pancreatic damage in type 1 diabetes.

Type 1 diabetes, a chronic autoimmune disorder in which progressive destruction or damaging of pancreatic β-cells in the islets of Langerhans, has in common high blood glucose levels (hyperglycemia) that can cause serious health complications [32]. The chronic hyperglycemia is associated with long-period damage, dysfunction, and failure of various organs, especially the eyes, heart, blood vessels, kidneys, and nerves. The most commonly used agent for the induction of type 1 diabetes is STZ. STZ, an antibiotic produced by *Streptomyces chromogenes*, liberates toxic amounts of NO, which is known to be destructive to pancreatic islet cells [1]. In addition, STZ enters the β-cell via glucose transporter and causes alkylation of DNA in pancreatic islet cells. This series of process result in the formation of O_2_^−^, ∙OH and H_2_O_2_, and intensify insulitis with accumulation of inflammatory cells and degranulation of pancreatic β-cells [33]. Actually, patients and rodents with diabetes experienced clinical signs such as polyuria, polydipsia, polyphagia, hunger, weight loss, and blurred vision [34, 35]. In our study, body weight reduced considerably and increased significantly food intake and water intake after the induction of diabetes. However, body weight of AH-treated rats was significantly increased compared to the diabetic control rats, although AH had no effect on food and water intakes. Also, AH administration reduced markedly the pancreatic weight to the normal level. These results suggest that AH enhanced body weight and decreased the pancreatic weight due to protecting from damage of the pancreatic tissue. In type 1 diabetes, destruction of pancreatic β-cells is believed to be mainly carried out by cytokines released from infiltrating lymphocytes and macrophages abnormally activated by an autoimmune response against various self-β-cell antigen in genetically susceptible subjects [36]. Both apoptosis and necrosis have been reported to be responsible for cell death in pancreatic β-cells exposed to immune and inflammatory cytokines [37] that can induce the expression of various pro-inflammatory and pro-apototic genes, essentially through the concomitant activation of the signal transducer and activator of transcription-1 (STAT-1) and the nuclear factor kB (NF-кB). A deficiency or insufficiency of insulin secretion caused by the injury of pancreatic islet cells shows high glucose in serum. In our experimental condition, diabetic rats showed the elevated glucose level and decreased insulin level. The destruction of β-cell and subsequent reduction in the islet mass observed in the H and E stained section of the diabetic untreated rats’ pancreas in a confirmation of the cytotoxic activity of STZ. This is as a result of increased rate of generation of ROS in the pancreas. Administration of AH extract to diabetic rats tends to repair the derangement caused to the pancreas by STZ. This is indicated by the regeneration of some β-cells and subsequent stimulation of insulin secretion. Therefore, it may be that AH protected the pancreatic β cells by decreasing oxidative stress and preserving pancreatic β cell integrity.

According to the HPLC profile, major components of AH were identified as protocatechuic acid and sulfur compound such as *S*-allyl cysteine and alliin (S-allyl cysteine sulfoxide, SACS). Protocatechuic acid (3,4-dihydroxybenzoic acid), a natural phenolic compound, is a major benzoic acid derivative with an excellent antioxidative effect, 10-fold higher than that of α-tocopherol. In addition, protocatechuic acid, a main anthocyanin metabolite, exerts anti-hyperglycemic effect on STZ-induced diabetic rats and anti-inflammatory effect on various diseases [38–40]. Alliin and its reduced form, *S*-allyl-cysteine well-known as the main active component of garlic absorbed in the intestine via the amino acid transported for cysteine. Thereafter, these increases blood insulin concentrations and improved glycemic control in diabetic rats [41–43]. Alliinase catalzed alliin to allicin (diallylthiosulfinate) and allicin also lowered the blood glucose during chronic hyperglycemia and suppressed free radical production [44]. As a matter of fact, AH may improve hyperglycemic state by the compositions including protocatechuic acid, S-allyl cysteine, and S-allyl cysteine sulfoxide is related to the regulation of insulin secretion. Hyperglycemia induced mitochondrial dysfunction and endoplasmic reticulum stress, promote ROS accumulation and ultimately leading to increased oxidative stress.

Oxidative stress is known to be increased in diabetes, since increased glucose increases oxidant production and impairs antioxidant defense system by multiple mechanisms, including increased intracellular formation of ROS [45]. The excessive production of ROS leads to an imbalance of the antioxidant system and finally causes cell and tissue damages. In the present study, AH administration to diabetic rats significantly attenuated oxidative stress by reducing ROS in serum and pancreas. These results could suggest that the effect of AH effectively influenced both the control of oxidative stress-induced pancreatic damage and serum glucose adjustment by insulin. Recent evidence has underlined the emerging role of HO-1 which is a rate-limiting enzyme in heme degradation processes in diabetes [46]. HO-1 induced by heavy metals, cytokines, UV light, oxidative stress, inflammatory cytokines and many drugs, and catalyzed the degradation of heme to biliverdin, iron, and so on. Herein, biliverdin is rapidly converted to bilirubin. Induction of HO-1 removes pro-oxidant heme and produces CO and bilirubin which are vasoactive and anti-oxidant molecules [47]. The elevated levels of HO-1 improve the oxidative stress and cell survival will be related to diabetes. Our present data also showed that HO-1 protein levels were decreased in diabetic rats, but the levels were significantly increased by AH administration. Under physiological conditions Nrf2 locates in the cytoplasm and binds to its inhibitor kelch-like ECH-associated protein 1 (KEAP1). Upon exposure of cells to oxidative stress or electrophilic compounds, Nrf2 is free from KEAP1 and translocates into the nucleus to bind to antioxidant-responsive elements (ARE) in the genes encoding antioxidant enzymes such as NADPH quinoneoxidoreductase (NQO1), heme oxygenase-1 (HO-1), glutathione *S*-transferase, superoxide dismutase (SOD), catalase, and γ-glutamylcysteine synthetase, increasing their expression to play a role in the detoxification, antioxidant, and anti-inflammatory [48]. Nrf2 is appreciated now for its potential prevention of or therapy for diabetic complications [49].

Hyperglycemia-induced oxidative stress or activation of other pathways may cause tissue damage. It could activate some oxidant-sensitive intracellular signaling pathways such as the induction of NF-кB transcription factor. NF-кB is a ubiquitous transcription factor that regulates the inflammatory response and whose overexpression seems to be related to type 1 diabetes. Accordingly, a significantly higher nuclear localization of p65 was seen in our type 1 diabetic patients, supporting the role of this important transcription factor in the atheromatous process through the increase of proinflammatory cytokines [38]. Inflammation represents a protective response that suppresses infections and accelerates tissue repair, but it can also contribute to local tissue damage in an extensive influence of inflammatory disorders. The inflammatory responses are associated with variations of plasma proteins and pro-inflammatory cytokines [50]. Pro-inflammatory cytokines, TNF-α and IL-6, are produced during the inflammatory process and are principal stimulators of acute-phase proteins and other markers of chronic inflammation commonly detected in diabetes mellitus [51]. In addition, IL-6 is a pleiotropic cytokine related with the regulation of the immune response, inflammation, and hematopoeisis [52]. TNF-α and IL-6 appear in the early phase of the inflammatory response and play an important role in the pathophysiology of inflammation in pancreatic tissue [30, 53]. In the present study, the elevated protein expression of NF-кB and its-related inflammatory cytokines (TNF-α and IL-6) in the pancreas of type 1 diabetic rats were downregulated significantly by the administration of AH, suggesting that AH can adjust inflammatory response by inhibiting the NF-кB pathway (Fig. 7).

Histological examination in pancreas revealed that pancreatic cells from AH-treated rats were less damaged (no vacuolization and less degranulation) compared with those in diabetic control rats. On the basis of these results, we suggest that AH delays the progression of diabetes through the inhibition of pancreatic injury.

## Conclusion

The present study indicates that AH may improve the pancretic β-cell damage and exhibit pancreatic anti-inflammatory activity through a reduction of hyperglycemia and/or inhibition of NF-кB-related pro-inflammatory cytokine expressions via the up-regulation of HO-1 protein expression in pancreas of STZ-induced type 1 diabetic rats. AH showed a new therapeutic possibility through improvement of insulin reduced in type 1 diabetic rats. Therefore, the current study suggests that AH could exert its pancreato-protective potential through the inhibition of oxidative stress-sensitive mechanisms of the pro-inflammatory response in the type 1 diabetics.
